# Supplementary figures and images for: Group A Streptococcal asparagine metabolism regulates bacterial virulence
Source: EMBO Rep. 2025 Apr 14;26(10):2767–91. doi: 10.1038/s44319-025-00447-z (PMC12117059; doi:10.1038/s44319-025-00447-z)

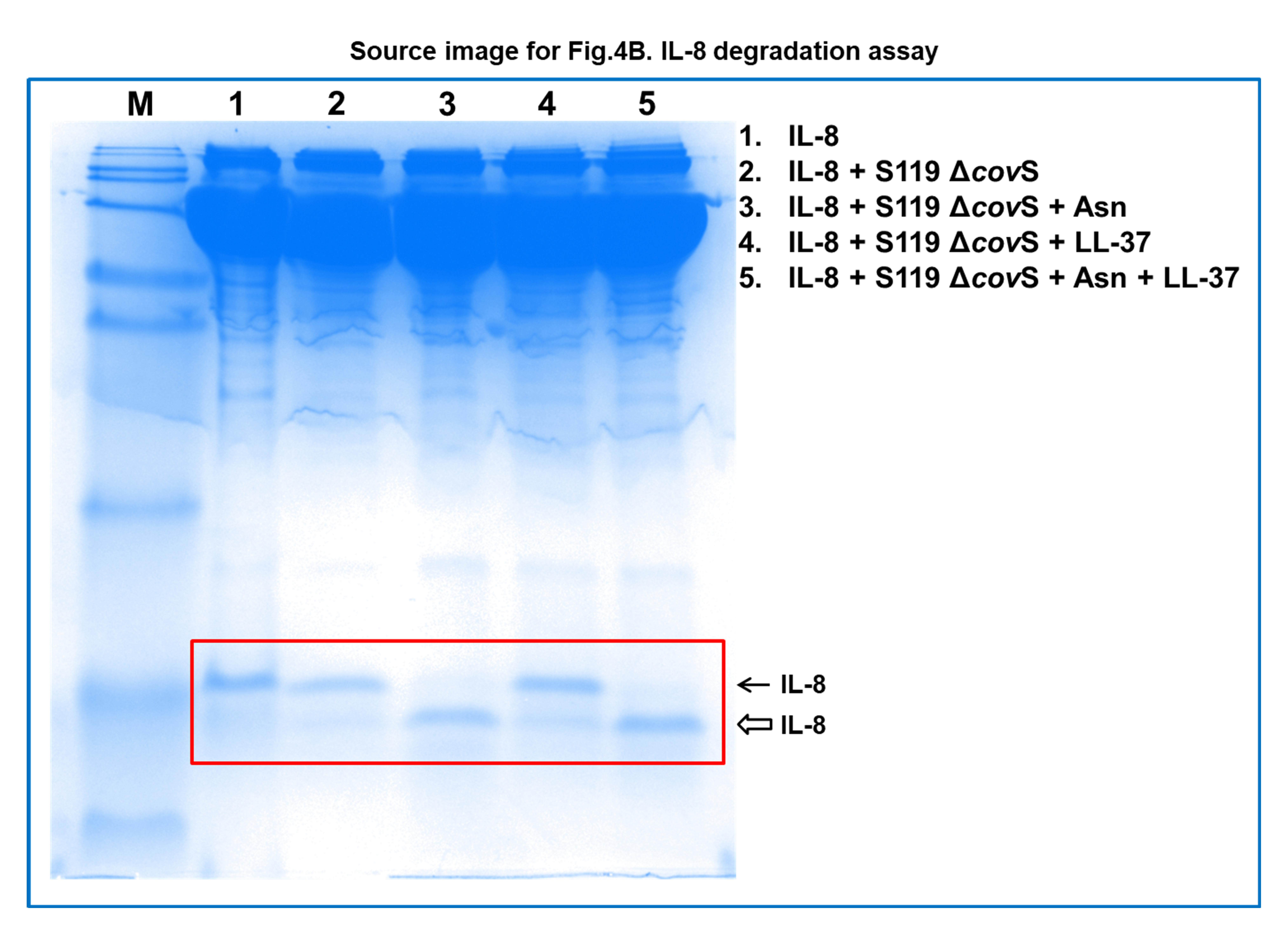

Supplement: Supplementary file 5 — Source data Fig. 4 [file 44319_2025_447_MOESM5_ESM.zip › SD.Fig.4B.jpg]

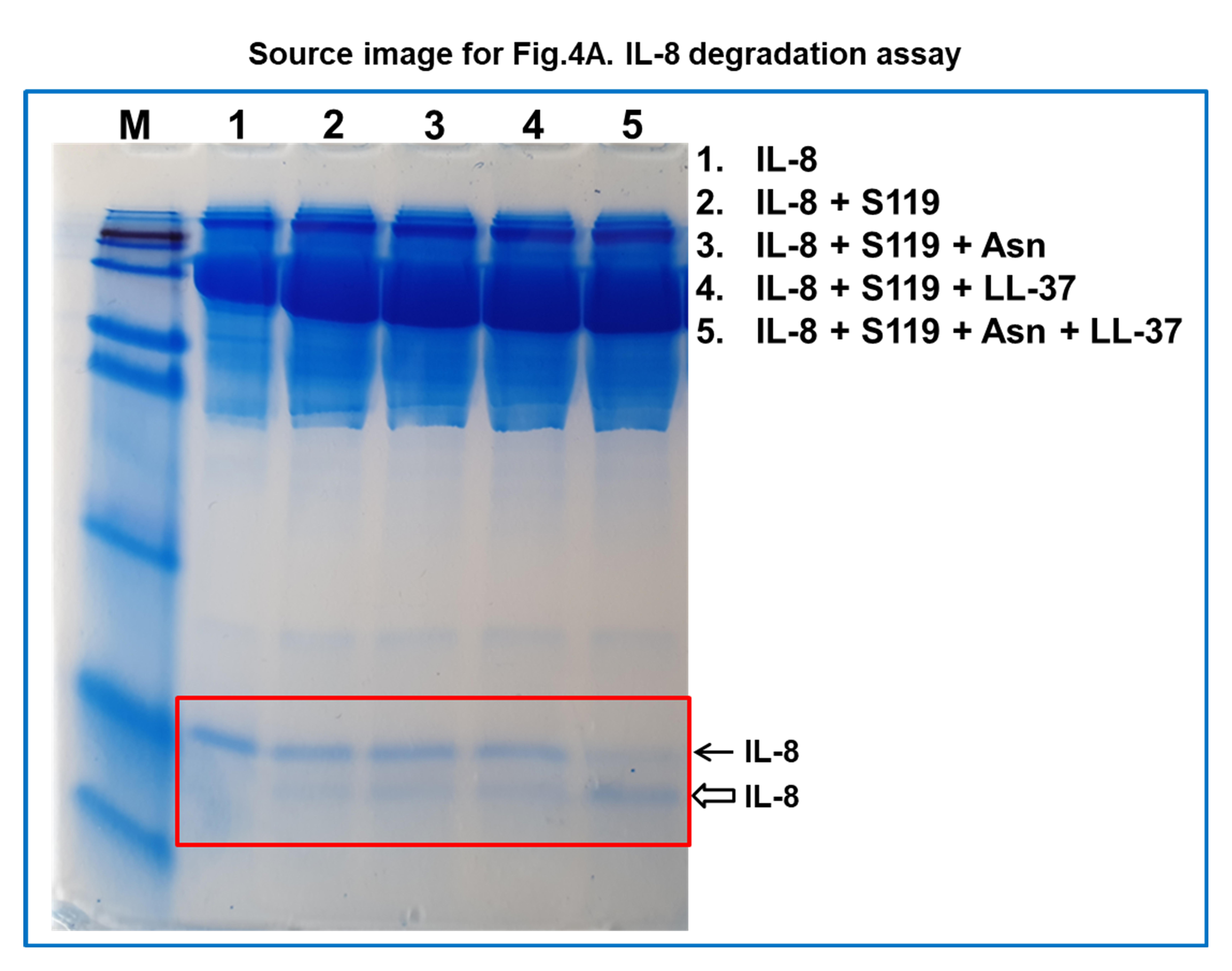

Supplement: Supplementary file 5 — Source data Fig. 4 [file 44319_2025_447_MOESM5_ESM.zip › SDFig.4A.jpg]
