# Supplementary material for: Group A Streptococcal asparagine metabolism regulates bacterial virulence
Source: EMBO Rep. 2025 Apr 14;26(10):2767–91. doi: 10.1038/s44319-025-00447-z (PMC12117059; doi:10.1038/s44319-025-00447-z)
Supplement: Supplementary file 9 — Expanded View Figures [file 44319_2025_447_MOESM9_ESM.pdf]

Expanded View Figures

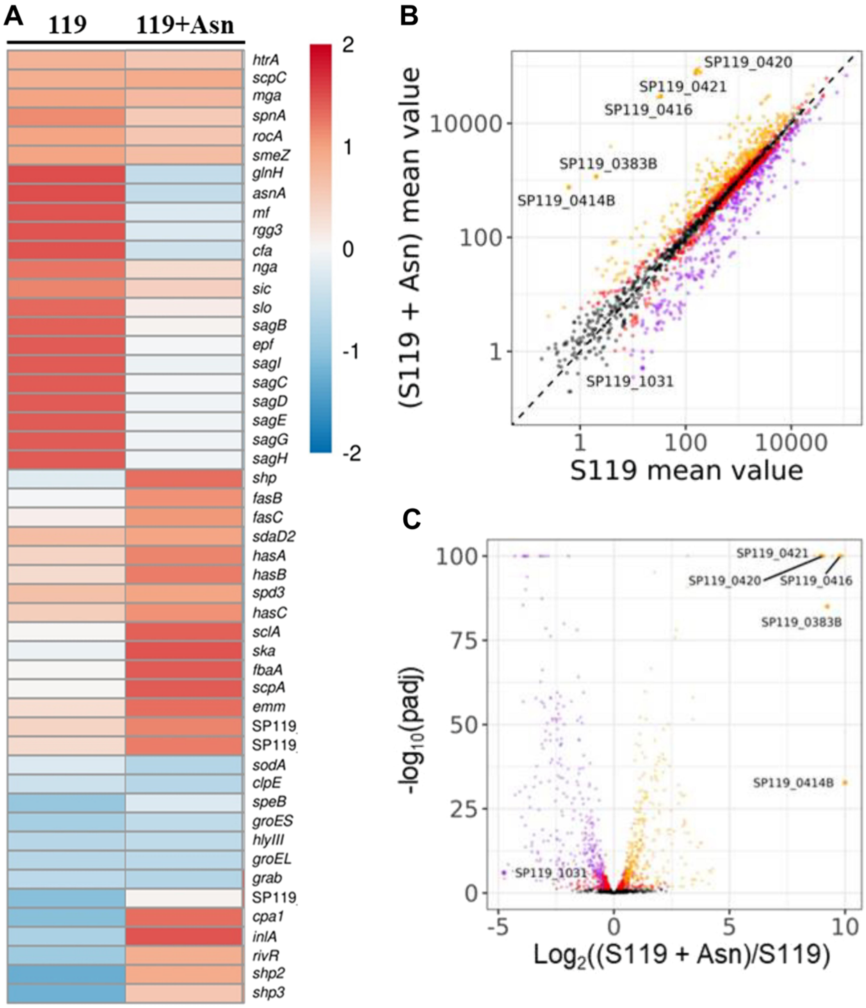

Figure EV1. Asn regulates GAS transcriptome.

(**A**) The heatmap represents the expression profile of genes, including virulence factors regulated by CovR/S directly or indirectly. (**B**, **C**) Visualization of mRNA-seq data. (**B**) A scatterplot matrix of the RNAseq data. Each dot represents a normalized mean value of the transcript number of the gene. (**C**) A Volcano plot of the same data set; each dot represents a gene with adjusted $P < 0.05$. as in Fig. 1. Data information: Four biological replicates were used (**A–C**).

© The Author(s)

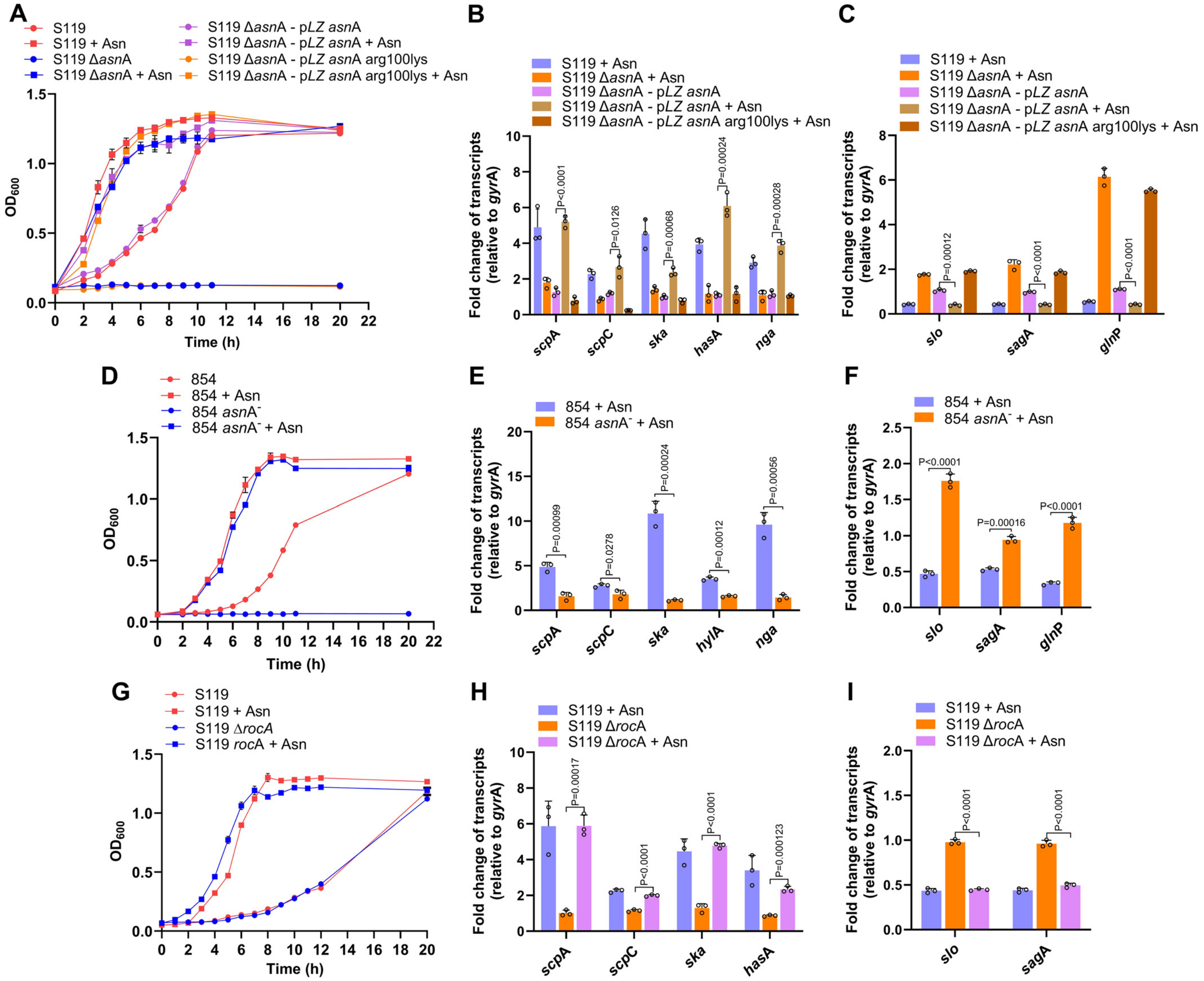

Figure EV2. *asnA* is essential for Asn-mediated gene regulation.

(**A**) The growth curves of stains S119, S119Δ*asn*A, S119Δ*asn*A-p*LZasn*A, and S119Δ*asn*A-p*LZasn*A arg100lys. (**B**, **C**) qRT-PCR determinations for Set 1 (**B**) and Set 2 (**C**) were conducted as described in Fig. 2. (**D**) The growth of the GAS 854 strain and its *asn*A⁻derived mutant was monitored in CDM at indicated times. (**E**, **F**) qRT-PCR determinations for genes of Set 1 (**E**) and genes of Set 2 (**F**) were determined as described in Fig. 1. (**H**, **I**) RocA is not involved in Asn-mediated effects. The growth (**G**) and qRT-PCR determinations for Set 1 (**H**) *slo* and *sag*A, and (**I**) for S119 and its Δ*roc*A-derived mutant were performed as described above. Data information: Three biological replicates were used (**A–I**). The data shown represent the means ± SD. Statistical analysis was performed using an unpaired two-tailed *t* test (**B**, **C**, **E**, **F**, **H**, **I**). Source data are available online for this figure.

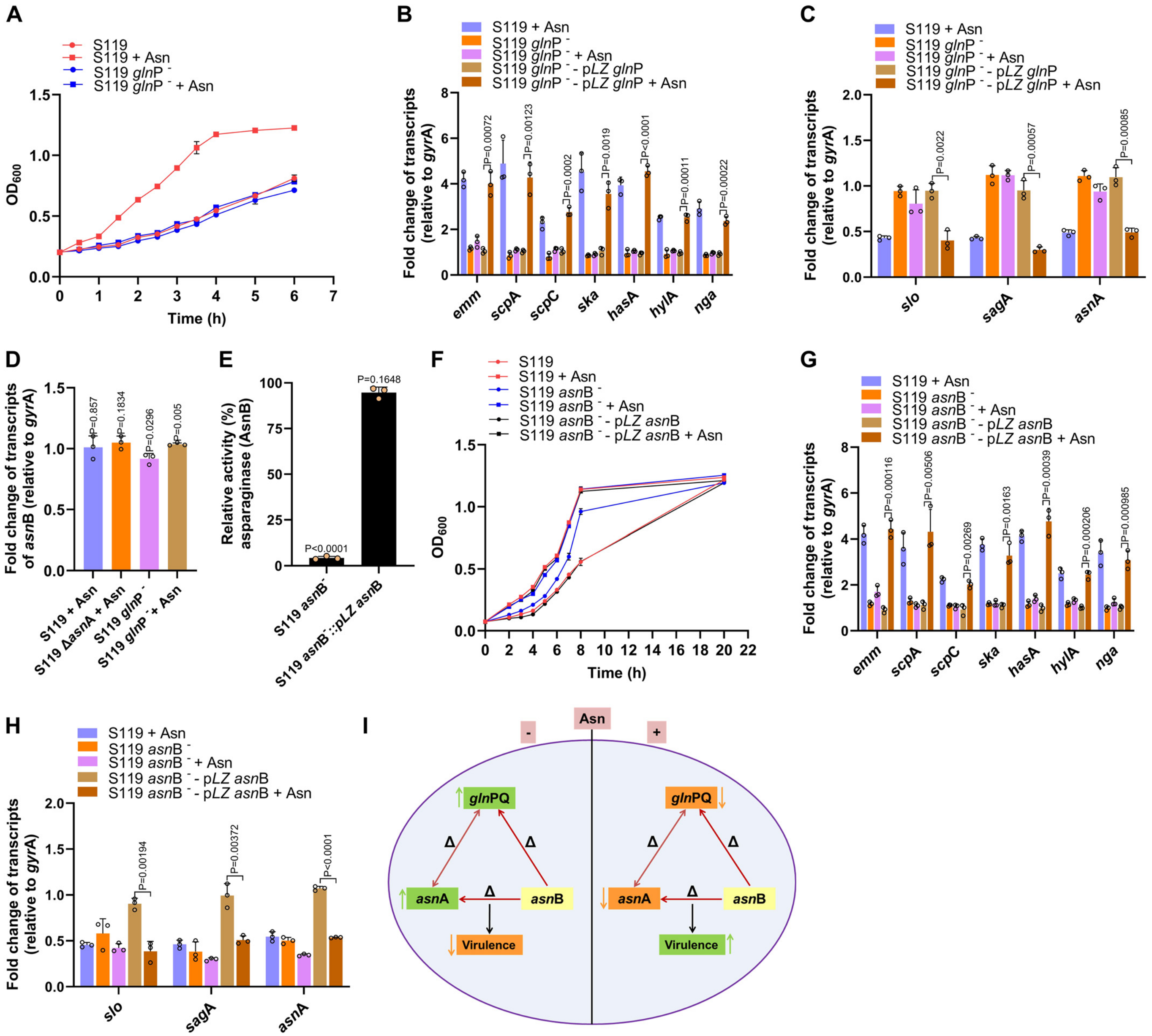

Figure EV3. *gln*P and *asn*B are essential for Asn-mediated gene regulation.

(**A**) The growth of S119 and S119 *gln*P⁻ in the absence or presence of Asn was determined (see Fig. 3B for the experimental details). (**B**, **C**) qRT-PCR determinations were conducted on Set 1 (**B**) or Set 2 of genes (**C**) using the indicated strains in CDM without or with Asn. (**D**) The *asn*B transcript is unaffected by the presence or absence of Asn or by the deficiency of AsnA or GlnPQ activities. qRT-PCR determinations of *asn*B transcript abundance were conducted as in (**B**, **C**). (**E**) Asparaginase activity (AsnB) was determined in the cell pellets of the indicated strains grown in THY. The asparaginase activity of WT S119 was used as a control. (**F**) The growth of the indicated strains was monitored in CDM in the absence or presence of Asn (10 and 100 μg ml^{-1}). (**G** and **H**) qRT-PCR determinations of Set 1 (**G**) and Set 2 (**H**), genes of the indicated strains grown in CDM without or with Asn. For all qRT-PCR data, fold change was calculated by comparing with normalized transcript abundance in the GAS S119 strain without Asn. (**I**) A graphical representation summarizes the orchestrated interplay between *asn*A, *gln*P, and *asn*B genes to maintain the balance of intracellular Asn in GAS. Data information: Three biological replicates were used (**A**–**H**). The data shown represent the means ± SD. Statistical analysis was performed using an unpaired two-tailed *t* test (**B**–**E**, **G**, **H**). Source data are available online for this figure.

 © The Author(s)

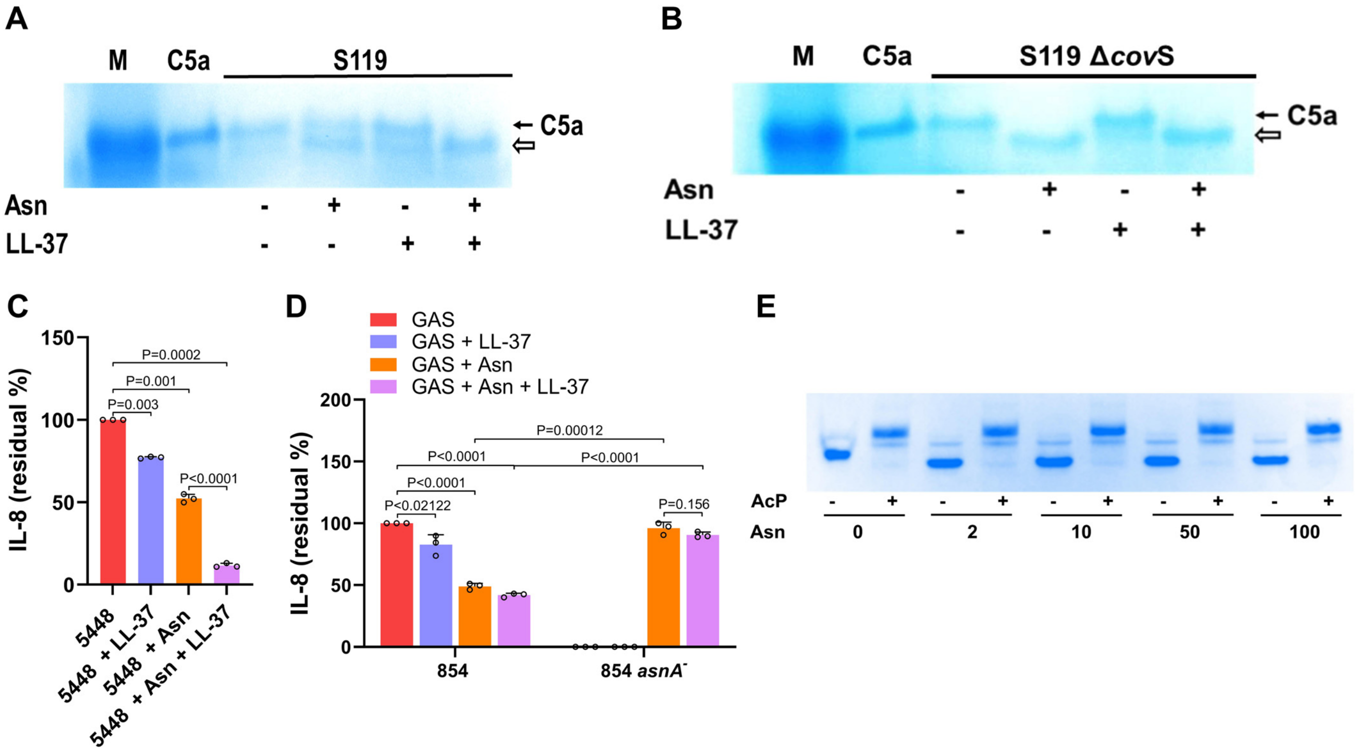

Figure EV4. Functional assays and in-vitro phosphorylation of CovR.

(**A**, **B**) Degradation of recombinant C5a by cultures supernatant containing the C5a-peptidase enzyme (ScpA). GAS was grown in CDM without or with Asn or/and LL-37. Supernatants of the indicated strains were incubated with recombinant human C5a, resolved on Tris-tricine gels, and visualized by Coomassie blue staining. M represents a marker, and an empty arrow is the cleaved C5a. The data are representative of two independent experiments. (**C**, **D**) Quantitation of IL-8 degradation by ScpC present in the supernatants of the indicated strains by ELISA. The IL-8 residual content in all supernatants was normalized to that of the GAS 5448 (**C**) and 854 (**D**) strain without Asn. (**E**) Asn does not affect in-vitro CovR phosphorylation. Purified CovR was incubated in the absence (−) and presence (+) of acetyl phosphate (Ac-P) as a phosphate donor and the indicated concentrations of Asn (μg ml^{-1}). The protein samples were resolved on Phos-tag SDS-PAGE gel and visualized. Data information: Three biological replicates were used (**C**, **D**). The data shown represent the means ± SD. Statistical analysis was performed using an unpaired two-tailed *t* test (**C**, **D**). Source data are available online for this figure.

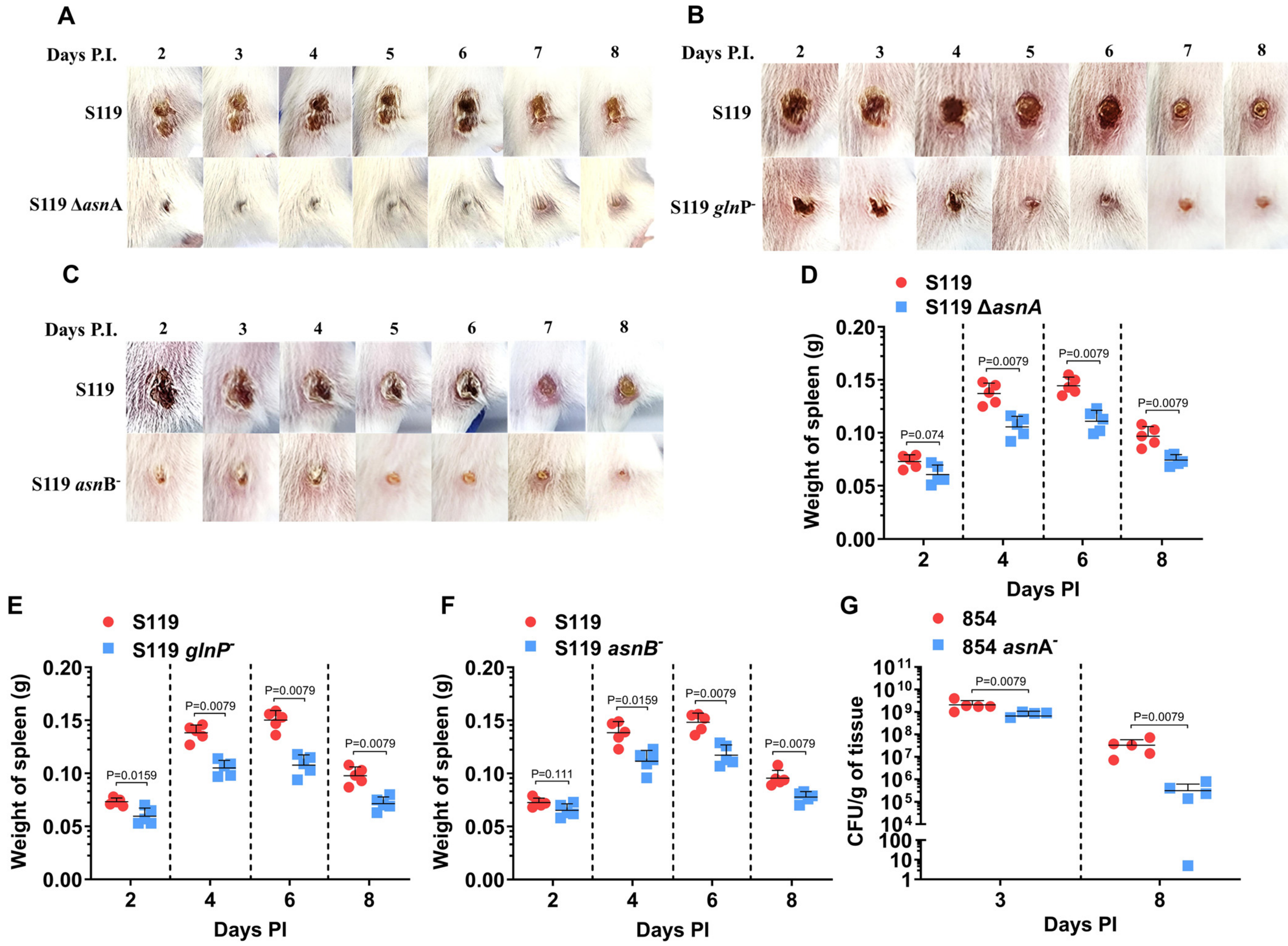

Figure EV5. ***asn*****A, *gln*****P, and *asn*****B mutants are attenuated in the sublethal murine model of human NF.**

(**A–C**) Mice were injected subcutaneously, and representative images of lesion progression at indicated time points after infection with S119 Δ*asn*A (**A**), S119 *gln*P⁻ (**B**), and S119 *asn*B⁻ (**C**), compared to WT S119 are shown. (**D–F**) Spleen weight of mice infected subcutaneously with S119 Δ*asn*A (**D**), S119 *gln*P⁻ (**E**), and S119 *asn*B⁻ (**F**), in comparison to S119, was determined. (**D–G**) The deletion of *asn*A produces an attenuated mutant in GAS strain 854. Mice were infected subcutaneously, and CFU counts per gram of soft tissue infected with 854 *asn*A⁻ compared to the wild-type, 854 were enumerated at indicated time intervals. Data information: Five mice per group per data point were used (**D–G**). The values shown represent the means ± SD. Statistical analysis was performed using the Mann–Whitney *U* test (**D–G**). Source data are available online for this figure.

 © The Author(s)

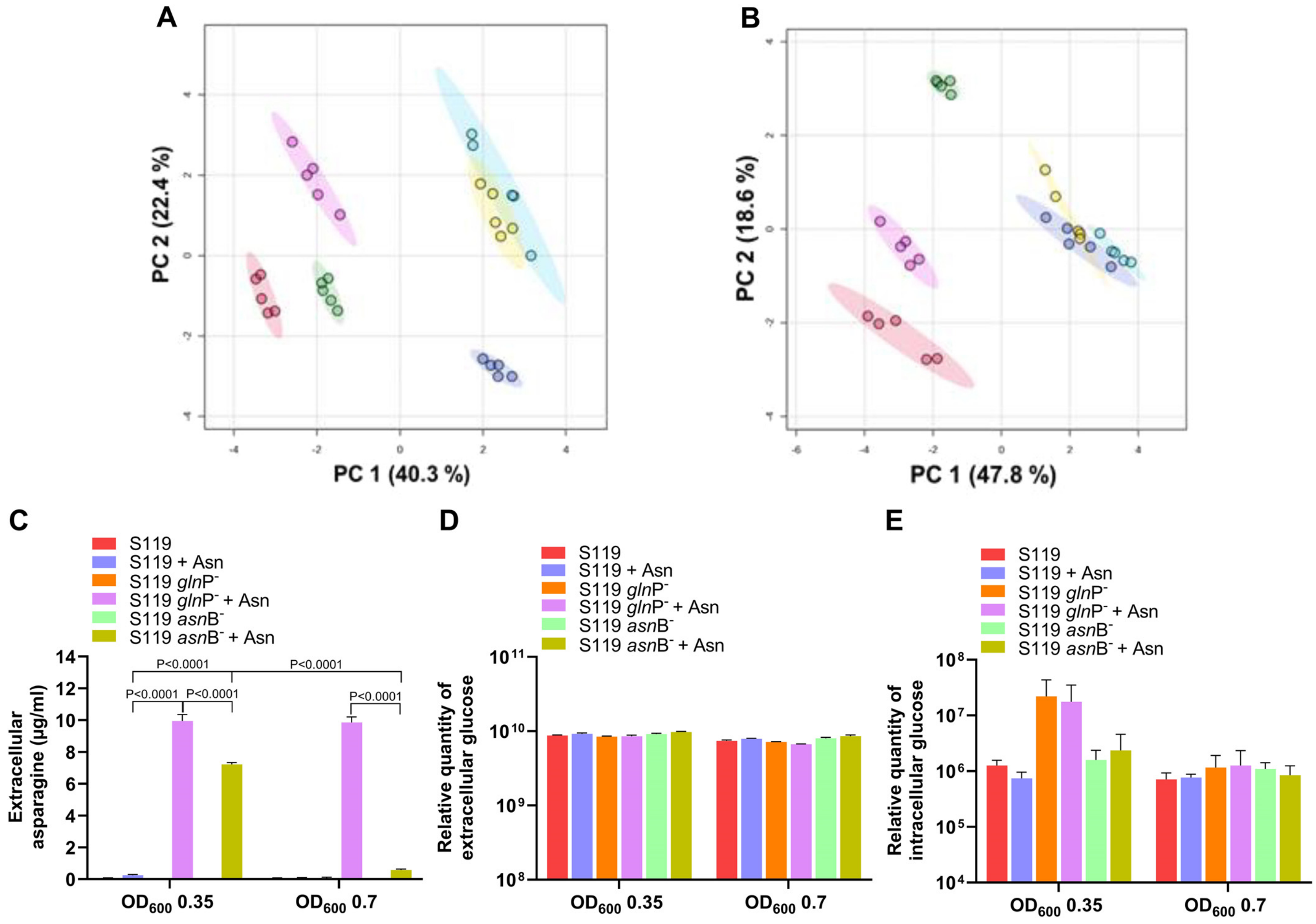

Figure EV6. Coupling of metabolism to virulence.

(**A**, **B**) The probabilistic principal component analyses (PCA) for the indicated pairs of strains grown in CDM or CDM supplemented with Asn to $OD_{600} = 0.35$ (**A**) or 0.7 (**B**). (**C**, **D**) Each circle represents one sample. Pink and yellow indicate S119 with and without Asn, respectively; blue and sky blue S119 *glnP*⁻ with and without Asn; and red and green S119 *asnB*⁻ with and without Asn. (**C**, **D**) Extracellular metabolite, Asn (**C**) content was determined for the indicated strains grown in CDM or CDM-supplemented with Asn to $OD_{600} = 0.35$ or 0.7. (**D**) The relative amount of glucose was determined for the indicated strains grown in CDM or CDM supplemented with Asn at $OD_{600} = 0.35$ or 0.7. (**E**) The relative amount of intracellular glucose was determined as above at $OD_{600} = 0.35$ or 0.7. Data information: Five biological replicates were used (**A**–**E**). The values shown represent the means ± SD. Statistical analysis was performed using an unpaired two-tailed *t* test (**C**–**E**). Source data are available online for this figure.
